# Supplementary material for: Fast, accurate antibody structure prediction from deep learning on massive set of natural antibodies
Source: Nat Commun. 2023 Apr 25;14:2389. doi: 10.1038/s41467-023-38063-x (PMC10129313; doi:10.1038/s41467-023-38063-x)
Supplement: Supplementary file 3 — Reporting Summary [file 41467_2023_38063_MOESM3_ESM.pdf]

nature portfolio

Corresponding author(s): Jeffrey J. Gray

Last updated by author(s): Mar 8, 2023

Reporting Summary

Nature Portfolio wishes to improve the reproducibility of the work that we publish. This form provides structure for consistency and transparency in reporting. For further information on Nature Portfolio policies, see our Editorial Policies and the Editorial Policy Checklist.

Statistics

For all statistical analyses, confirm that the following items are present in the figure legend, table legend, main text, or Methods section.

n/a	Confirmed	
☐	☒	The exact sample size (*n*) for each experimental group/condition, given as a discrete number and unit of measurement
☒	☐	A statement on whether measurements were taken from distinct samples or whether the same sample was measured repeatedly
☐	☒	The statistical test(s) used AND whether they are one- or two-sided<br>*Only common tests should be described solely by name; describe more complex techniques in the Methods section.*
☒	☐	A description of all covariates tested
☒	☐	A description of any assumptions or corrections, such as tests of normality and adjustment for multiple comparisons
☐	☒	A full description of the statistical parameters including central tendency (e.g. means) or other basic estimates (e.g. regression coefficient) AND variation (e.g. standard deviation) or associated estimates of uncertainty (e.g. confidence intervals)
☐	☒	For null hypothesis testing, the test statistic (e.g. *F*, *t*, *r*) with confidence intervals, effect sizes, degrees of freedom and *P* value noted<br>*Give P values as exact values whenever suitable.*
☒	☐	For Bayesian analysis, information on the choice of priors and Markov chain Monte Carlo settings
☒	☐	For hierarchical and complex designs, identification of the appropriate level for tests and full reporting of outcomes
☒	☐	Estimates of effect sizes (e.g. Cohen's *d*, Pearson's *r*), indicating how they were calculated

Our web collection on statistics for biologists contains articles on many of the points above.

Software and code

Policy information about availability of computer code

Data collection	RepertoireBuilder: current version from https://sysimm.org/rep_builder/ as of Sept 4, 2022<br>DeepAb: current version from https://github.com/rosettaCommons/deepab as of Nov 22, 2022<br>ABlooper: version 1.1.2<br>ColabFold: version 1.3.0<br>NanoNet: current version from https://github.com/dina-lab3D/NanoNet as of Aug 31, 2022
Data analysis	PyRosetta: version 2022.12+release.a4d7970

For manuscripts utilizing custom algorithms or software that are central to the research but not yet described in published literature, software must be made available to editors and reviewers. We strongly encourage code deposition in a community repository (e.g. GitHub). See the Nature Portfolio guidelines for submitting code & software for further information.

Data

Policy information about availability of data

All manuscripts must include a data availability statement. This statement should provide the following information, where applicable:

- Accession codes, unique identifiers, or web links for publicly available datasets
- A description of any restrictions on data availability
- For clinical datasets or third party data, please ensure that the statement adheres to our policy

The structure prediction data used for benchmarking in this study have been deposited in the Zenodo database under accession code 10.5281/zenodo.7677723 [https://doi.org/10.5281/zenodo.7677723]. Paired antibody structures predicted by IgFold for the 104 thousand OAS sequences and 1.3 million human sequences are available at https://github.com/Graylab/IgFold. Experimentally determined structures used for model training and evaluation were accessed from the Protein Data Bank (PDB) [https://www.rcsb.org]. Natural antibody sequences used for data augmentation were accessed from the Observed Antibody Space (OAS) [https://opig.stats.ox.ac.uk/webapps/oas/].

Human research participants

Policy information about studies involving human research participants and Sex and Gender in Research.

Reporting on sex and gender	The present study did not involve human participants.
Population characteristics	The present study did not involve human participants.
Recruitment	The present study did not involve human participants.
Ethics oversight	The present study was not subject to ethics oversight.

Note that full information on the approval of the study protocol must also be provided in the manuscript.

Field-specific reporting

Please select the one below that is the best fit for your research. If you are not sure, read the appropriate sections before making your selection.

☒ Life sciences ☐ Behavioural & social sciences ☐ Ecological, evolutionary & environmental sciences

For a reference copy of the document with all sections, see nature.com/documents/nr-reporting-summary-flat.pdf

Life sciences study design

All studies must disclose on these points even when the disclosure is negative.

Sample size	Structure samples for benchmarking were selected by collecting all antibodies deposited in the Protein Data Bank after July 1, 2021. This date cutoff ensures that no methods evaluated had access to the structures during training. The resulting structures were de-duplicated at 99% sequence identity to avoid biasing the benchmark results towards identical structures. Any structures with resolution less than 3.0 Å were discarded to ensure only reliable structures were used for analysis. Any structures with CDR loops longer than 20 residues were discarded to more closely match the natural CDR loop length distribution and not bias prediction results with outliers (which tend to be poorly modeled). These steps resulted in 197 paired and 71 single-chain antibody structures for benchmarking methods. These sample sizes are sufficient to observe a diverse range of loop lengths and conformations, and represent all of the data available at the time of collection.
Data exclusions	No data were excluded from the analyses.
Replication	Model training was successfully replicated four times.
Randomization	Randomization is not relevant to this study. All structures were predicted by each method and the results were analyzed to assess performance.
Blinding	Blinding is not relevant to the present study. Investigators accessed distinct data partitions for model training and evaluation, but were aware of which partitions had been designated for each task.

Reporting for specific materials, systems and methods

We require information from authors about some types of materials, experimental systems and methods used in many studies. Here, indicate whether each material, system or method listed is relevant to your study. If you are not sure if a list item applies to your research, read the appropriate section before selecting a response.

Materials & experimental systems

n/a	Involved in the study
☒	☐ Antibodies
☒	☐ Eukaryotic cell lines
☒	☐ Palaeontology and archaeology
☒	☐ Animals and other organisms
☒	☐ Clinical data
☒	☐ Dual use research of concern

Methods

n/a	Involved in the study
☒	☐ ChIP-seq
☒	☐ Flow cytometry
☒	☐ MRI-based neuroimaging
